# LGR5 expression predicts peritoneal recurrence after curative resection of primary colon cancer

**DOI:** 10.1038/s41416-019-0442-5

**Published:** 2019-04-19

**Authors:** Hiroshi Nagata, Soichiro Ishihara, Hiroyuki Abe, Tetsuo Ushiku, Junko Kishikawa, Toshiaki Tanaka, Keisuke Hata, Kazushige Kawai, Masashi Fukayama, Hiroaki Nozawa

**Affiliations:** 1 Department of Surgical Oncology, The University of Tokyo, Tokyo, Japan; 2 Department of Pathology, The University of Tokyo, Tokyo, Japan

**Keywords:** Predictive markers, Colon cancer, Metastasis

## Abstract

**Background:**

The aim of this study was to clarify whether a cancer stem cell marker could be an indicator of post-operative peritoneal recurrence of colon cancer.

**Methods:**

Expression of four putative markers (CD133, CD44 variant 6, aldehyde dehydrogenase-1 and leucine-rich repeating G-protein-coupled receptor-5 (LGR5)) was evaluated immunohistochemically in primary tumour samples from 292 patients who underwent curative resection for non-metastasised pT4 colon cancer at the University of Tokyo Hospital between 1997 and 2015.

**Results:**

Peritoneal recurrence was significantly higher in LGR5-negative cases (5-year cumulative incidence: 27.5% vs. 14.4%, *p* = 0.037). Multivariable analysis confirmed that negative LGR5 expression was an independent risk factor for peritoneal recurrence (hazard ratio (HR) 2.79, *p* = 0.005) in addition to poor differentiation, positive lymph node metastasis, preoperative carcinoembryonic antigen > 5 ng/mL and anastomotic leakage. The addition of LGR5 significantly improved the predictive value of the multivariable model (net reclassification improvement: 0.186, *p* = 0.028: integrated discrimination improvement: 0.047, *p* = 0.008).

**Conclusions:**

Negative LGR5 expression was a significant predictor of peritoneal recurrence in patients with pT4 colon cancer. Therefore, LGR5 might be a promising biomarker to identify patients at high risk of post-operative peritoneal metastasis.

## Background

Despite significant advances in treatments for colorectal cancer, the prognosis of patients with peritoneal metastasis remains dismal.^1^ Although the prognosis is strongly affected by the extent of carcinomatosis, detection of the disease in its early stage is still challenging due to the poor diagnostic sensitivity of current imaging modalities. In view of these difficulties, aggressive preventive measures against post-operative peritoneal metastasis were advocated, including upfront hyperthermic intraperitoneal chemotherapy or a systematic second-look operation.^2,3^ High-risk patients may benefit from the interventions, whereas patient selection is the key.

In order to improve the risk assessment, we conceived that the expression of a cancer stem cell marker might be an indicator of peritoneal recurrence. The cancer stem cell is defined as ‘a cell within a tumour that possesses the capacity to self-renew and to cause the heterogeneous lineages of cancer cells that comprise the tumour.’^4^ This type of cell is considered to play a significant role in tumour progression and metastatic dissemination in various types of cancers,^5^ and be responsible for the resistance of cancer tissues towards treatment. We assumed that peritoneal metastasis was compatible with the characteristics of cancer stem cells because tumour initiation and proliferation abilities are required to form each peritoneal nodule, and they are known to be resistant to chemotherapy.

Since there is no definitive determinant of cancer stem cells, we evaluated the expression of four putative stem cell markers, namely, CD133, CD44 variant 6 (CD44v6), aldehyde dehydrogenase-1 (ALDH1) and leucine-rich repeating G-protein-coupled receptor-5 (LGR5) and investigated whether they were effective clinical biomarkers to identify patients with a high risk of post-operative peritoneal metastasis. To our knowledge, this is the first attempt to elucidate the impact of the expression of cancer stem cell markers on peritoneal recurrence.

## Methods

### Patients and tissue specimens

This is a historical cohort study which included patients who underwent curative resection for non-metastatic pT4 colon cancer without preoperative chemotherapy at the University of Tokyo Hospital (Tokyo, Japan) between 1997 and 2015. We excluded patients with perforated tumours, because perioperative rupture of primary tumour can be an independent indication of proactive management.^2^ Consequently, we included 292 patients in the final analysis, and retrospectively retrieved data regarding their clinicopathological characteristics, treatments and clinical outcomes from their medical records. Median follow-up time of these patients was 63.6 months. We focused on patients with pT4 disease, a well-known risk factor of peritoneal recurrence,^2,3,6^ to ensure that a sufficient number of events were evaluated to determine the clinical impacts of cancer stem cell markers on patient prognosis.

All patients were clinically staged using physical examination, colonoscopy and chest–abdomen–pelvis computed tomography (CT). No evidence of peritoneal metastasis was found during the primary tumour resection. Pathological staging of the primary tumours was performed according to the Union for International Cancer Control TNM Classification of Malignant Tumours, eighth edition.^7^ Tumours proximal to the hepatic flexure were classified as right colon cancer, and those distal to the splenic flexure were classified as left colon cancer. Post-operative surveillance was performed for 5 years as follows: tumour marker testing every 3 months, chest–abdomen–pelvis CT every 6 months and colonoscopy every 12 months.^8^ Systematic second-look operations were not performed.

The study protocol was approved by the research ethics committee at the Graduate School of Medicine, the University of Tokyo (3252-(7)). The research was conducted in accordance with the 1964 Declaration of Helsinki and its later amendments. In addition, this study followed Reporting recommendations for tumour marker prognostic studies (REMARK) statement.

### Immunohistochemical evaluation

Consecutive 3-μm formalin-fixed paraffin-embedded sections of the invasive front of the primary tumour were used for the evaluation by immunohistochemistry (IHC). As previously described,^9^ antigen retrieval was performed in 10 mM sodium citrate buffer (pH 6.0) for 5 min at 120 °C using an autoclave. Incubation with a primary antibody was performed at 4 °C overnight for CD133 (clone AC133; 1:100 dilution; Miltenyi Biotec, Auburn, CA, USA),^10^ CD44v6 (clone VFF-18; 1:1000 dilution; Abcam, Cambridge, MA, USA),^11^ ALDH1 (clone EP1933Y; 1:100 dilution; Abcam, Cambridge, MA, USA)^12^ and LGR5 (LS-A1232; 1:400 dilution; LifeSpan Biosciences, Seattle, WA, USA).^13^ After the incubation with a Dako Envision Kit (Dako, Carpinteria, CA, USA), Meyer’s haematoxylin (Sigma Chemical Co., St. Louis, MO, USA) was used for counterstaining. For the positive control, renal tubules, skin tissues, liver tissues and the crypt base of the normal colon mucosa were used for CD133, CD44v6, ALDH1 and LGR5, respectively. For the negative control, the antibody was replaced with phosphate-buffered saline.

Expression was defined as positive when CD133, CD44v6, ALDH1 and LGR5 staining was found in more than 5%, 25%, 25% and 5% of the epithelium part of the tumour in accordance with previous reports.^10,13–15^ The evaluations were performed independently by two observers, including at least one pathologist. Evaluators were blinded to the clinical findings, and discrepancies were resolved by discussion.

### In situ hybridisation

Sections at 7 µm from the same blocks as immunohistochemical staining were used for in situ hybridisation (ISH) to confirm the expression of LGR5. After deparaffinisation, sections were fixed with 10% neutral buffered formalin, followed by 0.2% HCl for 10 min at 37 °C before digestion with proteinase K solution (10 μg/ml) for 10 min at 37 °C. Hybridisation was performed using digoxigenin-labelled RNA probe against LGR5 (Genostaff) for 16 h at 60 °C at concentrations of 250 ng/ml. Thereafter, sections were incubated with an anti-digoxigenin antibody conjugated with alkaline phosphatase (Roche, Indianapolis, IN, USA) diluted at a ratio of 1:2000 for 1 h at room temperature. Colouring reactions were performed overnight with nitroblue tetrazolium/5-bromo-4-chloro-3-indolyl-phosphate solution (Sigma-Aldrich). Sections were counterstained with Kernechtrot stain solution (Muto PureChemicals, Tokyo, Japan).

### Statistical analysis

Categorical variables were described using frequencies and percentages. Correlation was evaluated using a chi-squared test or Fisher’s exact test. The distributions of continuous variables were described using medians and the interquartile ranges. The Mann–Whitney test was used for comparison. Patient age at primary tumour resection was categorised as < 65 years or ≥ 65 years. Tumour size was categorised as ≥ 50 mm or < 50 mm,^16^ and the CEA level was categorised on the basis of the upper normal limit (5 ng/ml) as ≤ 5 ng/ml or > 5 ng/ml.

Overall survival was defined as the time from the primary tumour resection to death due to any cause, and relapse-free survival was defined as the duration between the date of the surgery and the first documented recurrence or death from any cause, whichever occurred first. The time to post-operative peritoneal metastasis was defined as the duration between the date of curative resection of the primary tumour and the date of clinical diagnosis of peritoneal metastasis as the first site of post-operative recurrence.

Survival data were calculated using Kaplan–Meier method and compared using the log-rank test. Univariable and multivariable Cox regression analyses were performed to investigate patient and tumour characteristics associated with the risk of post-operative peritoneal metastasis. Only variables with significant differences in the univariable analysis were included in the multivariable analysis. Through the time-to-event analysis, hazard ratios (HRs) and 95% confidence intervals (CIs) were generated.

The performance of a multivariable Cox hazard model was evaluated using area under the curve (AUC) at 60 months, and Harrell’s c-index.^17^ Net reclassification improvement (NRI) and integrated discrimination improvement (IDI)^18^ were used to compare performances of models. Associations were considered significant for *P*-values < 0.05. Data were statistically analysed using the statistical program *R 3.5.1* (http://www.R-project.org/) using the following packages: *survival, timeROC* and *survIDINRI*. This study was a complete-case analysis, and missing data were not recognised within the predictors we evaluated.

## Results

### Immunohistochemical images of stem cell marker expression

**Fig. 1:**
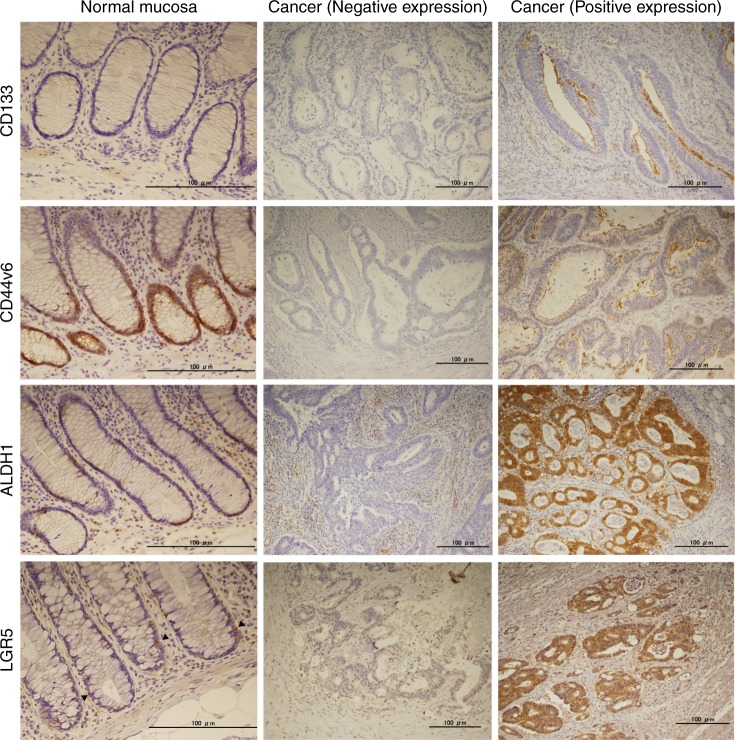
Representative images of cancer stem cell markers by immunohistochemistry in normal colonic tissue and colon cancer. LGR5-positive cells (arrow heads) are recognised at the bottom of a normal colonic crypt

The immunohistochemical staining patterns of CD133, CD44v6, ALDH1 and LGR5 are shown in Fig. 1. Membranous immunoreactivity was observed for the former two, and cytoplasmic immunoreactivity for the latter two. The expression of CD44v6, ALDH1 and LGR5 was recognised at the bottom of a normal colonic crypt, compatible with the site of stem cells.

### Clinicopathological characteristics and the expression of cancer stem cell markers in primary tumours

Clinicopathological characteristics of the 292 patients with available specimens are shown in Table 1. The 5-year overall and relapse-free survival rates in the whole patient group were 76.8% and 56.4%, respectively. The 5-year cumulative incidence of peritoneal recurrence in this patient group was 16.8%.

**Table 1:** Clinicopathological characteristics

*n* = 292		*N* (%)
Age	< 65 years	115 (39.4%)
	≥ 65 years	177 (60.6%)
Gender	Male	166 (56.8%)
	Female	126 (43.2%)
Site of primary tumour	Right	107 (36.6%)
	Left	185 (63.4%)
Tumour size	< 50 mm	137 (46.9%)
	≥ 50 mm	155 (53.1%)
Differentiation	Well/moderate	281 (96.2%)
	Poor	11 (3.8%)
Histology	Tubular adenocarcinoma	265 (90.8%)
	Mucinous adenocarcinoma	27 (9.2%)
Lymphatic invasion	ly0	163 (55.8%)
	ly1	129 (44.2%)
Venous invasion	v0	55 (18.8%)
	v1	237 (81.2%)
T category	T4a	236 (80.8%)
	T4b	56 (19.2%)
N category	N0	125 (42.8%)
	N1	121 (41.4%)
	N2	46 (15.8%)
Lymph node count	0–11	44 (15.1%)
	≥ 12	248 (84.9%)
Preoperative CEA, ng/mL	≤ 5 ng/mL	128 (43.8%)
	> 5 ng/mL	164 (56.2%)
Large bowel obstruction	Obstruction (−)	141 (48.3%)
	Obstruction ( + )	151 (51.7%)
The use of laparoscopy	Open surgery	202 (69.2%)
	Laparoscopic surgery	90 (30.8%)
Anastomotic leakage	Leak (−)	287 (98.3%)
	Leak ( + )	5 (1.7%)
Adjuvant chemotherapy	None	168 (57.5%)
	< 3 months	18 (6.2%)
	≥ 3 months	106 (36.3%)
CD133	CD133 (−)	119 (40.8%)
	CD133 ( + )	173 (59.2%)
CD44v6	CD44v6 (−)	79 (27.1%)
	CD44v6 ( + )	213 (72.9%)
ALDH1	ALDH1 (−)	158 (54.1%)
	ALDH1 ( + )	134 (45.9%)
LGR5	LGR5 (−)	53 (17.2%)
	LGR5 ( + )	239 (81.8%)

*ALDH1* aldehyde dehydrogenase-1, *CEA* carcinoembryonic antigen, *CD44v6* CD44 variant 6, *LGR5* leucine-rich repeating G-protein-coupled receptor-5

The positive rates of CD133, CD44v6, ALDH1 and LGR5 expression in the primary tumour samples were 59.2% (173/292), 72.9% (213/292), 45.9% (134/292) and 81.8% (239/292), respectively. Concordance among evaluators was generally favourable (Kappa values were 0.73, 0.82, 0.75 and 0.92, respectively).

### Cancer stem cell marker expression in primary tumours and patient survival

None of the examined markers were significantly associated with overall survival (CD133-positive: 76.6% vs. CD133-negative 77.2%, *p* *=* 0.846; CD44v6-positive: 77.4% vs. CD44v6-negative 75.4%, *p* = 0.744; ALDH1-positive: 75.1% vs. ALDH1-negative 78.1%, *p* = 0.657; LGR5-positive: 78.2% vs. LGR5-negative 70.0%, *p* = 0.551) (Supplementary Fig. 1).

The same was true for relapse-free survival (CD133-positive: 57.5% vs. CD133-negative 54.8%, *p* = 0.383; CD44v6-positive: 54.2% vs. CD44v6-negative 61.9%, *p* = 0.267; ALDH1-positive: 58.9% vs. ALDH1-negative 54.6%, *p* = 0.478; LGR5-positive: 57.3% vs. LGR5-negative 52.2%, *p* = 0.780) (Supplementary Fig. 2).

### Cancer stem cell marker expression in primary tumours and peritoneal recurrence

**Fig. 2:**
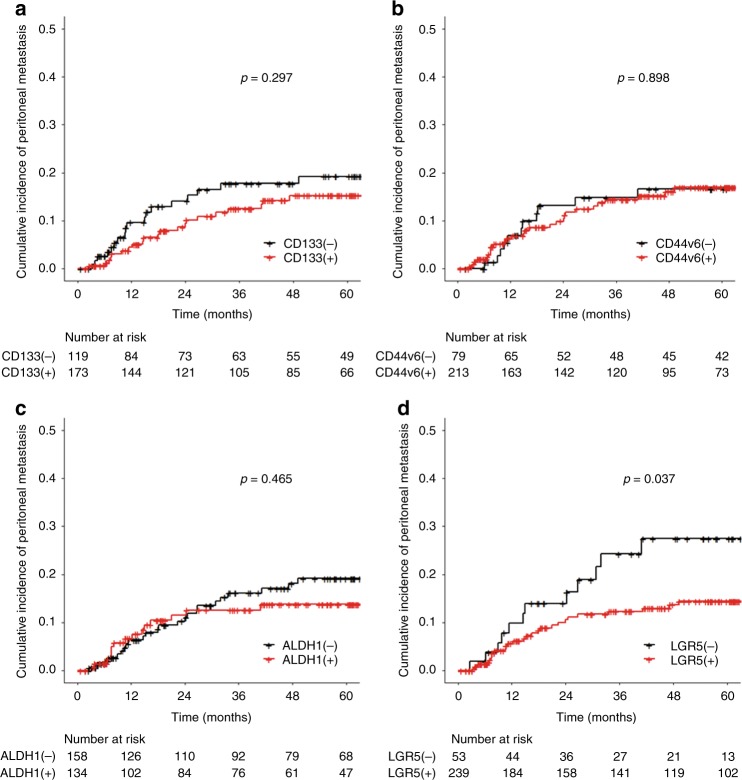
Stem cell marker expressions in primary tumour and time to peritoneal metastasis. CD133 (**a**), CD44v6 (**b**), ALDH1 (**c**) and LGR5 (**d**)

The incidence was significantly higher in LGR5-negative than LGR5-positive cases (27.5% vs. 14.4%, *p* = 0.037), whereas other markers were not associated with peritoneal recurrence (CD133-positive: 15.3% vs. CD133-negative 19.2%, *p* = 0.297; CD44v6-positive: 17.0% vs. CD44v6-negative 16.6%, *p* = 0.898; ALDH1-positive: 13.8% vs. ALDH1-negative 19.1%, *p* = 0.465) (Fig. 2).

Univariable and multivariable Cox regression analyses revealed that negative LGR5 expression was a statistically significant risk factor for peritoneal recurrence (HR: 2.79, 95% CI: 1.37–5.67, *p* = 0.005) as well as poor differentiation (HR: 3.90, 95% CI: 1.15–13.28, *p* = 0.029), positive lymph node metastasis (HR: 3.37, 95% CI: 1.44–7.86, *p* = 0.005), preoperative CEA > 5 ng/mL (HR: 2.25, 95% CI: 1.08–4.67, *p* = 0.031) and anastomotic leakage (HR: 8.57, 95% CI 1.81–40.68, *p* = 0.007) (Table 2).

**Table 2:** Risk factors for post-operative peritoneal metastasis after curative resection of pT4 colon cancer

	Univariable analysis			Multivariable analysis		
	HR	95% CI	*P*	HR	95% CI	*P*
Age (≥ 65 years)	0.65	0.35–1.22	0.183			
Gender (female)	0.84	0.44–1.61	0.606			
Site of primary tumour (left)	0.75	0.40–1.42	0.377			
Size (≥ 50 mm)	1.54	0.80–2.96	0.195			
Differentiation (poor)	3.65	1.13–11.91	0.032	3.90	1.15–13.28	0.029
Histology (mucinous adenocarcinoma)	1.84	0.72–4.71	0.203			
Lymphatic invasion (ly1)	2.06	1.09–3.90	0.027	1.32	0.65–2.67	0.442
Venous invasion (v1)	1.91	0.75–4.87	0.179			
T category (T4b)	0.55	0.20–1.55	0.256			
Lymph node metastasis	2.84	1.35–5.98	0.006	3.37	1.44–7.86	0.005
Lymph node count (≥ 12)	0.53	0.25–1.12	0.097			
Preoperative CEA (> 5 ng/ml)	2.10	1.05–4.22	0.037	2.25	1.08–4.69	0.031
Malignant bowel obstruction	1.43	0.78–2.71	0.268			
Laparoscopic surgery	1.24	0.65–2.39	0.516			
Anastomotic leakage	4.33	1.03–18.08	0.045	8.57	1.81–40.68	0.007
Adjuvant chemotherapy	1.26	0.67–2.37	0.481			
LGR5 expression (negative)	1.98	1.00–3.92	0.049	2.79	1.37–5.67	0.005

*CEA* carcinoembryonic antigen, *LGR5* leucine-rich repeating G-protein-coupled receptor-5

### Predictive value of LGR5

To determine the predictive value of LGR5, we evaluated the performance of the multivariable Cox hazard model with or without LGR5. The baseline model consisted of factors with significant differences in the univariable analysis: differentiation, lymphatic invasion, N category, preoperative CEA level and anastomotic leakage. This model exhibited an AUC of 0.73 at 60 months, and the c-index was 0.71. When LGR5 was added to the baseline model, AUC at 60 months significantly rose to 0.78 (*p* = 0.045), and the c-index was 0.74 (Table 3). NRI and IDI revealed significant improvements in discrimination (NRI: 0.195, *p* = 0.032 and IDI: 0.040, *p* = 0.022).

**Table 3:** Multivariable Cox models with or without LGR5 for postoperative peritoneal metastasis after curative resection of pT4 colon cancer

	A model without LGR5	A model with LGR5
*Derivation cohort model estimates*		
*N*	292	292
Number of events	39	39
Median follow-up time, months	63.6	63.6
	Beta	Beta
Differentiation		
Well/moderate	–	–
Poor	1.105	1.362
Lymphatic invasion		
ly0	–	–
ly1	0.311	0.278
N category		
N0	–	–
N1–2	1.095	1.215
Preoperative CEA level		
≤ 5 ng/mL	–	–
> 5 ng/mL	0.672	0.810
Anastomotic leak		
Leak (−)	–	–
Leak ( + )	2.054	2.148
LGR5		
LGR5 ( + )	–	–
LGR5 (−)	–	1.026
*Model assessment*		
AUC at 60 months	0.73	0.78
Concordance index	0.71	0.74

*CEA* carcinoembryonic antigen, *LGR5* leucine-rich repeating G-protein-coupled receptor-5

### Validation of the expression of LGR5 by in situ hybridisation

**Fig. 3:**
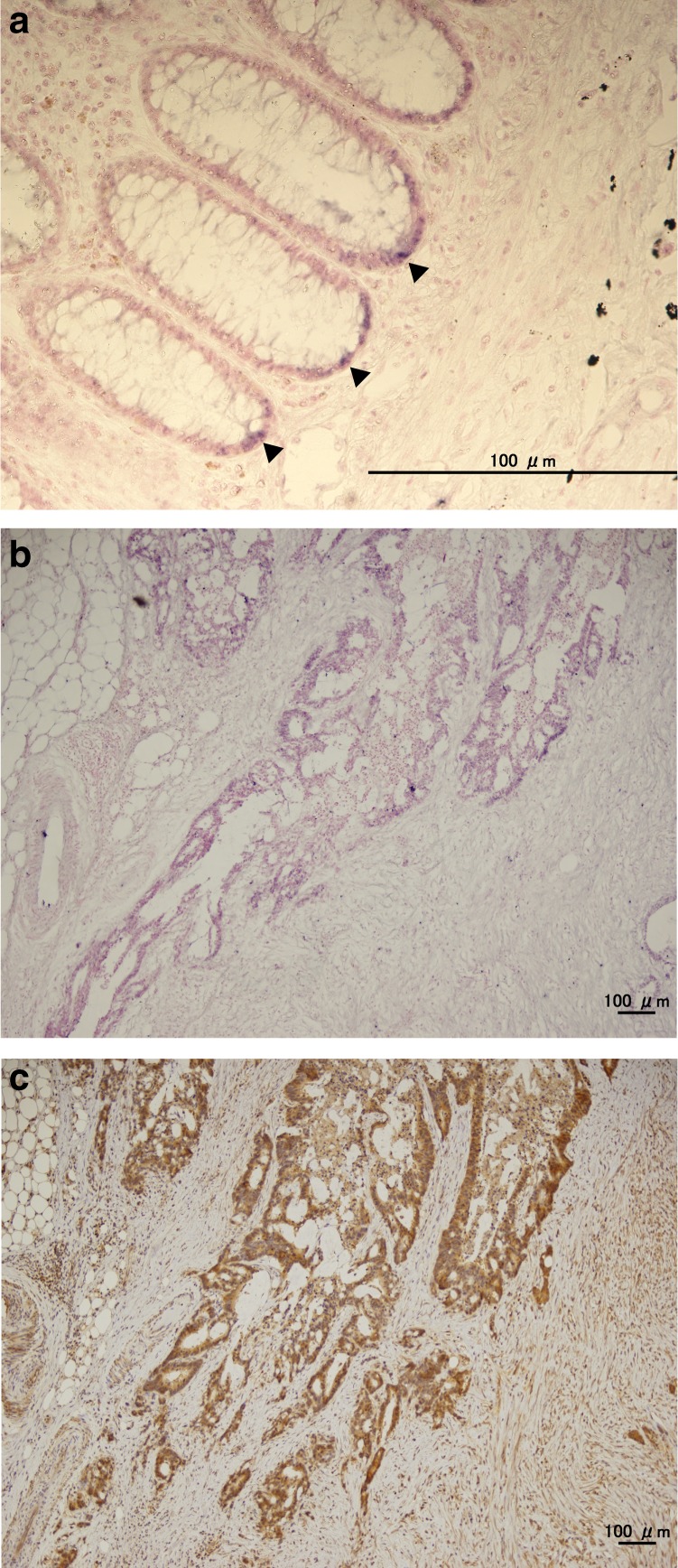
LGR5 expression by in situ hybridisation in normal colonic tissue (**a**) and colon cancer (**b**), and immunohistochemical staining of the corresponding cancer tissue (**c**). LGR5-positive cells (arrow heads) are recognised at the bottom of a normal colonic crypt

To confirm the expression of LGR5, in situ hybridisation (ISH) was also performed. As shown in Fig. 3, mRNA of LGR5 was recognised at the bottom of the normal crypt, and the distribution of ISH-positive cells was consistent with that of IHC-positive cells. We evaluated 57 tumour samples resected in 2014 and 2015 and found that 94.4% of the IHC-positive tumours were ISH-positive (34 out of 36), whereas 42.9% of IHC-negative tumours were ISH-positive (9 out of 21).

## Discussion

In this study, we investigated the expression of putative cancer stem cell markers in pT4 colon cancer and revealed that patients with LGR5-negative tumours had a higher risk of post-operative peritoneal metastasis. Moreover, we found that prediction of peritoneal recurrence could be significantly improved by factoring in the expression of LGR5. To the best of our knowledge, this is the first study to demonstrate that a stem cell marker can be a predictive biomarker of post-operative recurrence.

The association of a negative LGR5 expression with a higher risk of peritoneal recurrence that was observed in this study was contrary to our initial assumption. This result may appear to contradict the cancer stem cell theory; however, LGR5 is merely one of the putative markers of stemness, and its characteristics are not necessarily identical to those of cancer stem cells. Considering that the concordant prognostic impact was not recognised in the other three markers, we supposed the finding in this study is probably related to a molecular function unique to LGR5 rather than stemness.

LGR5 is a seven-transmembrane receptor that is widely recognised as a marker of intestinal stem cells.^19,20^ LGR5, when bound to R-spondin, can potentiate the Wnt/ β-catenin pathway by suppressing the negative feedback mechanism.^21,22^ However, the role of LGR5 in cancerous tissue is yet to be clarified.^23^ Based on the aforementioned function in a normal cell, LGR5 is presumed to promote tumour progression because the deregulation of Wnt/β-signalling is a fundamental inducer of colorectal cancer development. Indeed, a number of studies reported that LGR5 was associated with high proliferation activity, malignant transformation and treatment resistance in cancer.^24–27^ Nevertheless, studies have also reported that LGR5 can play a suppressive role in tumorigenesis.^23,28^ For example, de Sousa et al. reported that the LGR5 gene was silenced by CpG island methylation during tumorigenesis, and that its re-expression reduced tumour growth.^29^ Meanwhile, Wu et al. showed that, when binding to R-spondin 2, LGR5 imposes an inhibitory effect on canonical Wnt signalling.^22^ Regarding metastasis, Zhou et al. recently reported that LGR5 suppresses colon cancer metastasis by activating TGFβ signalling.^30^ Therefore, it is quite possible that LGR5-negative tumours exhibit a higher metastatic potential.

Furthermore, we found some studies, which indicated a possible link between LGR5 and peritoneal metastasis. LGR5 was reported to alter the actin cytoskeleton structure and increased cell–cell adhesion by coupling to the intracellular scaffold signalling protein.^28,31^ The study suggested that the major function of LGR5 may be to promote strong intracellular adhesion among stem cells and to retain them within the crypt base to maintain homoeostasis in the intestinal epithelium.^31^ Weakened adhesion between cancer cells may facilitate the formation of intraperitoneal-free cancer cells, which may lead to peritoneal metastasis. Hence, this finding may explain the potential of LGR-negative tumours to cause peritoneal metastasis.

One of the difficulties of research on stem cell markers is that the evaluation method varies widely for different studies and the results obtained can be inconsistent.^11^ We used 5% as a cut-off level for LGR5 based on a previous study on colorectal cancer using the same antibody.^13^ In order to ensure maximum credibility of the results, we used pathological sections which included not only the invasive front but also the normal colon tissue whenever possible, so that the normal part can be used as an internal control. In addition, evaluations of stains were independently performed by experts of the field. Furthermore, we confirmed the expression of LGR5 by in situ hybridisation. The results of IHC and ISH were not identical: the majority of the IHC-positive tumours were ISH-positive, while almost half of the IHC-negative tumours were ISH-positive. However, this gap can be rationally explained by post-transcriptional regulation: mRNA is not necessarily translated to protein by various mechanisms, including RNA splicing and RNA silencing.^32^ Therefore, we presume that the result of IHC in this study was fairly reasonable and reliable. We adopted IHC rather than ISH because IHC has the advantage of clinical practicability; the antibody is easily available and staining and evaluation can be performed by commonly known procedures.

Another limitation of this study is that the diagnosis of peritoneal metastasis was based on the findings of imaging. Despite the advancement of imaging modalities, the sensitivity for detecting small peritoneal nodules is still unsatisfactory.^33–35^ Hence, some patients classified as other sites of recurrence might have undetected peritoneal metastasis. While it may be ideal to perform a systematic second-look surgery for detecting peritoneal metastasis at an early stage,^2,3^ it is unrealistic to carry out surgical exploration for every patient without any signs of recurrence. Therefore, we believe, at this moment, it is feasible to utilise imaging studies to evaluate the likelihood of peritoneal recurrence.

In conclusion, this study revealed that the negative expression of LGR5 in primary tumours is a significant predictor of post-operative peritoneal metastasis in patients with non-metastatic pT4 colon cancer. LGR5 might be applicable as a clinical biomarker to identify patients who can benefit from aggressive management strategies against peritoneal recurrence of colon cancer.

## Supplementary information

Supplementary Figures

## Data Availability

The datasets generated and analysed during the current study are available from the corresponding author on reasonable request.
