# Supplementary material for: LGR5 expression predicts peritoneal recurrence after curative resection of primary colon cancer
Source: Br J Cancer. 2019 Apr 19;120(10):996–1002. doi: 10.1038/s41416-019-0442-5 (PMC6734652; doi:10.1038/s41416-019-0442-5)
Supplement: Supplementary file 1 — Supplementary Figures [file 41416_2019_442_MOESM1_ESM.docx]

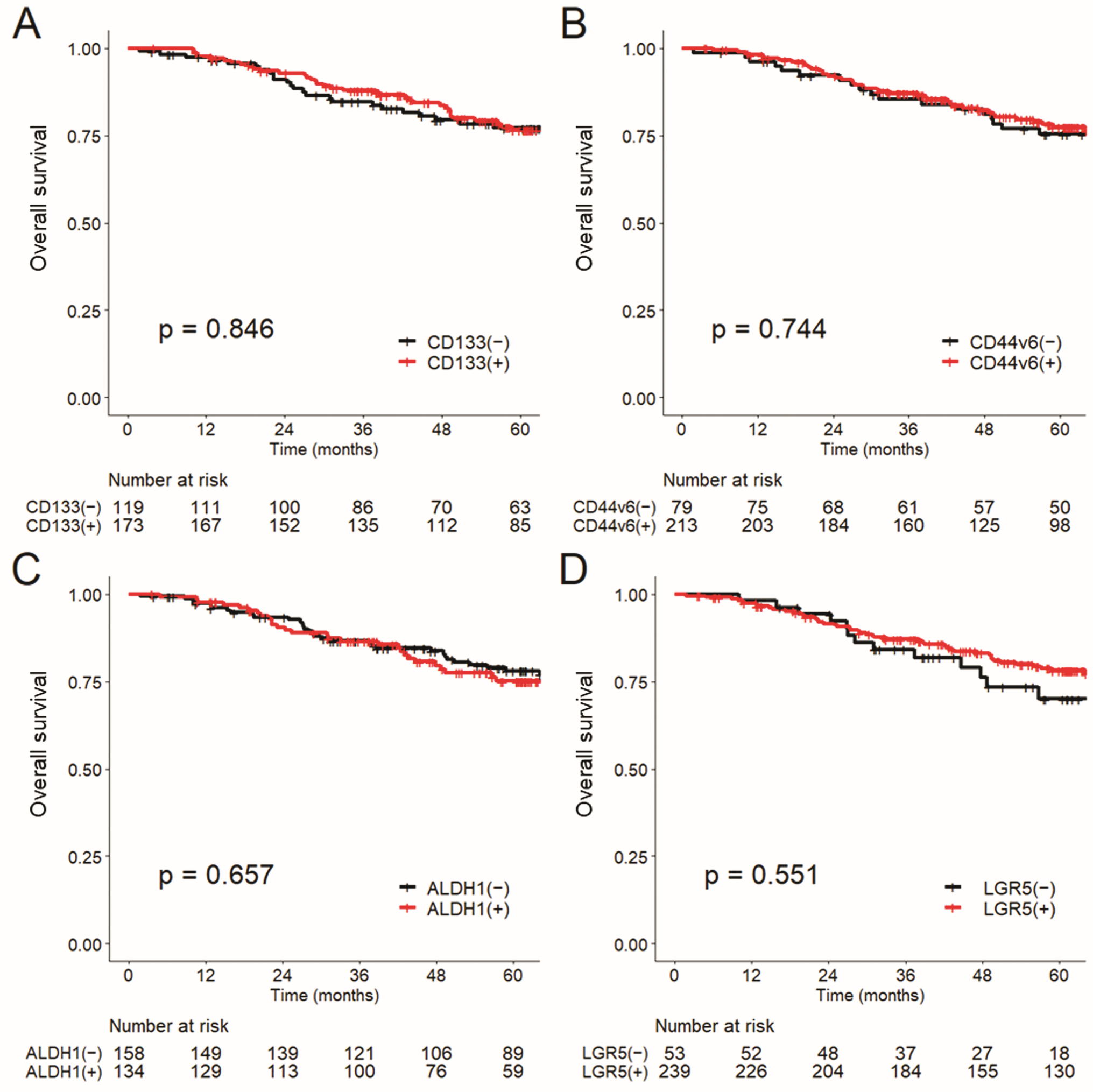


Supplementary Fig. 1: Stem cell marker expressions in primary tumor and overall survival.

CD133 (A), CD44v6 (B), ALDH1 (C), and LGR5 (D).


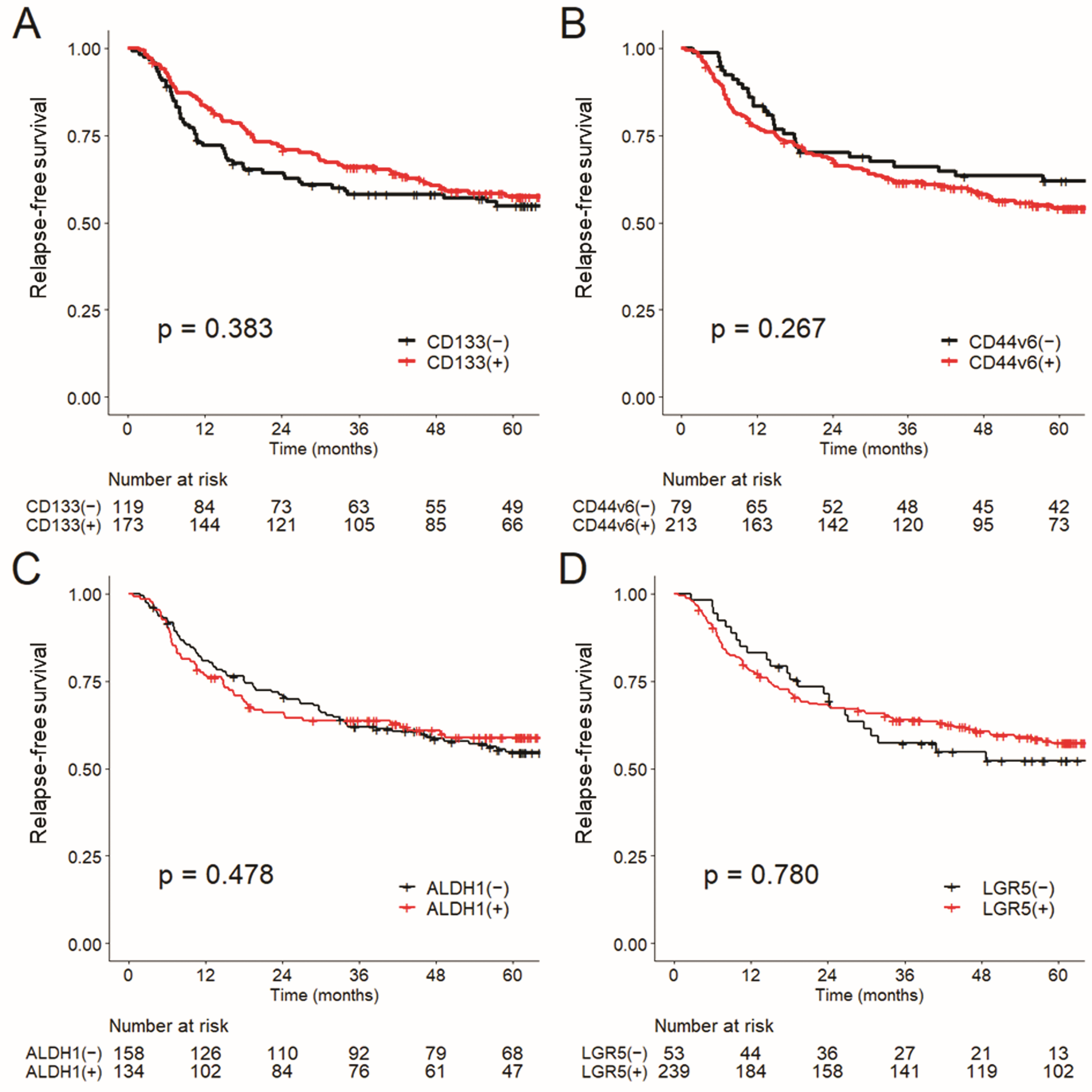


Supplementary Fig. 2: Stem cell marker expressions in primary tumor and relapse-free survival. CD133 (A), CD44v6 (B), ALDH1 (C), and LGR5 (D).
